# Final National and State-Level 2012–13 Influenza Vaccination Coverage Estimates Available Online

**Published:** 2013-09-27

Final state-specific influenza vaccination coverage estimates for the 2012–13 season are now available online at FluVaxView (http://www.cdc.gov/flu/fluvaxview). The online information includes estimates of the percentage of persons vaccinated during July 2012–May 2013, for each state, for each U.S. Department of Health and Human Services region, and for the United States overall.

Analyses were conducted using National Immunization Survey data for children aged 6 months–17 years and Behavioral Risk Factor Surveillance System data for adults aged ≥18 years. Estimates are provided by age group and race/ethnicity. These estimates are presented in an interactive report (http://www.cdc.gov/flu/fluvaxview/interactive.htm) with state-specific estimates and are complemented by an online summary report (http://www.cdc.gov/flu/fluvaxview/coverage-1213estimates.htm) with national estimates.

